# Supplementary material for: Morus alba Prevented the Cyclophosphamide Induced Somatic and Germinal Cell Damage in Male Rats by Ameliorating the Antioxidant Enzyme Levels
Source: Molecules. 2021 Feb 26;26(5):1266. doi: 10.3390/molecules26051266 (PMC7956634; doi:10.3390/molecules26051266)
Supplement: Supplementary file 1 [file molecules-26-01266-s001.pdf]

Supplementary Materials for Article

Morus alba Prevented the Cyclophosphamide Induced Somatic and Germinal Cell Damage in Male Rats by Ameliorating the Antioxidant Enzyme Levels

Abhijit Ghosh [1], Syed Imam Rabbani [1,2,*], Syed Mohammed Basheeruddin Asdaq [3,*], Yahya Mohzari [4], Ahmed Alrashed [5], Hamdan Najib Alajami [6], Awad Othman Aljohani [6], Abdullah Ali Al Mushtawi [6], Majed Sultan Alenazy [6], Rakan Fahad Alamer [6] and Abdulmajead Khalid Alanazi [6]

1 Department of Pharmacology, Al-Ameen College of Pharmacy, Bangalore-560027, India
2 Department of Pharmacology and Toxicology, College of Pharmacy, Qassim University, Buraydah 51452, Saudi Arabia
3 Department of Pharmacy Practice, College of Pharmacy, AlMaarefa University, Dariyah, Riyadh 13713, Saudi Arabia
4 Clinical pharmacy department, King Saud Medical City, Riyadh 12746, Saudi Arabia
5 Pharmaceutical services administration, Inpatient Department, main hospital, KFMC, Riyadh 11564, Saudi Arabia
6 Pharmaceutical Services Administration, King Saud Medical City, Ministry of Health, Riyadh 12746, Saudi Arabia

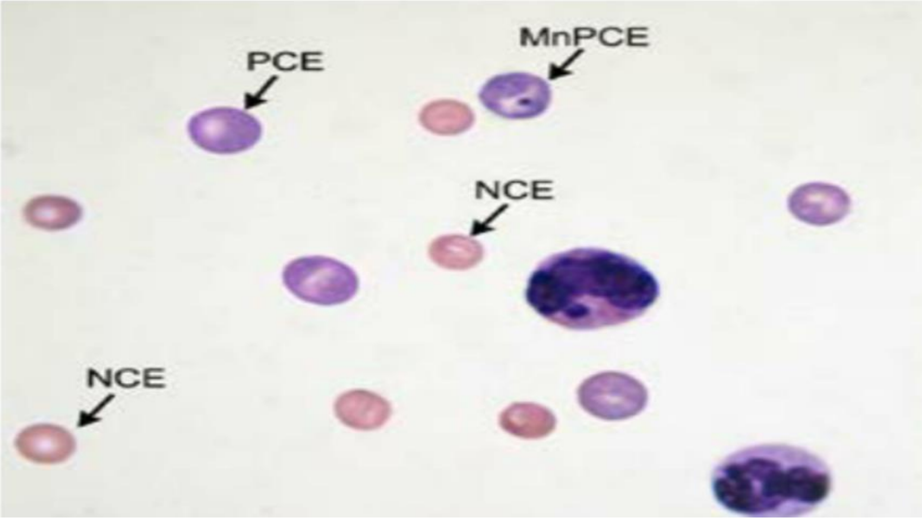

Figure S1. Photographs showing the micronuclei in bone marrow erythrocytes.

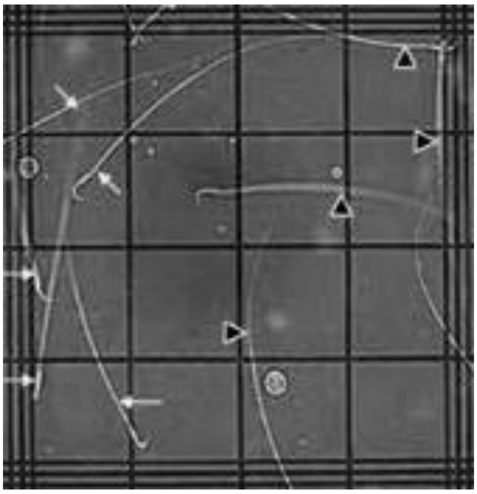

Figure S2. Photographs showing the sperm count using Neubauers' chamber.

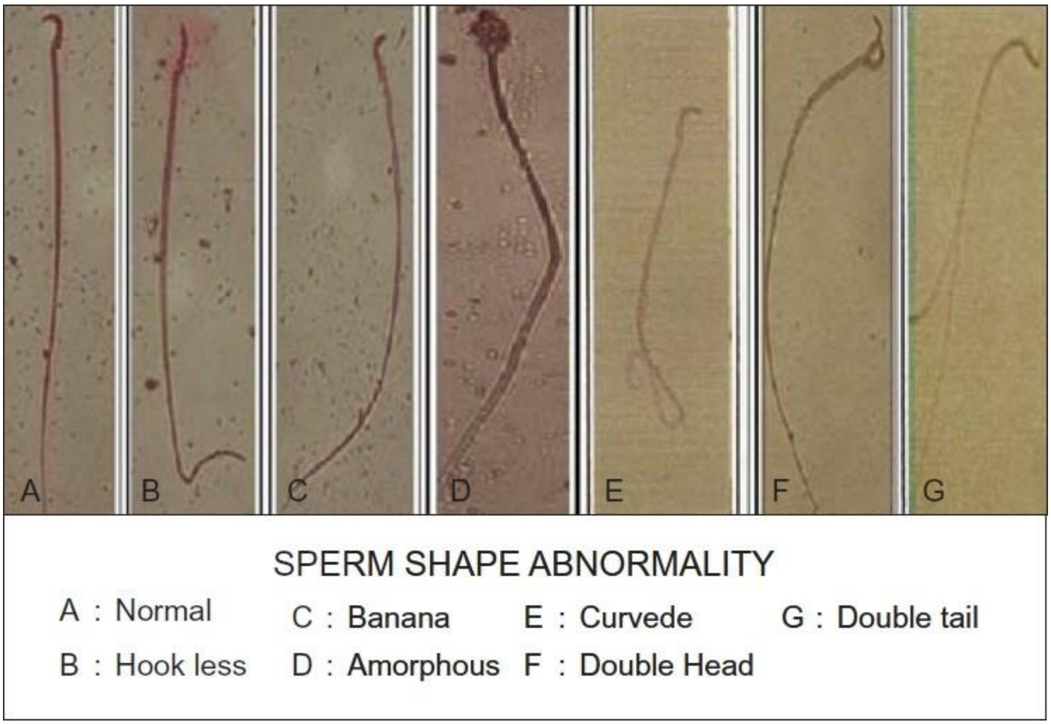

Figure S3. Photographs showing the sperm shape abnormalities.

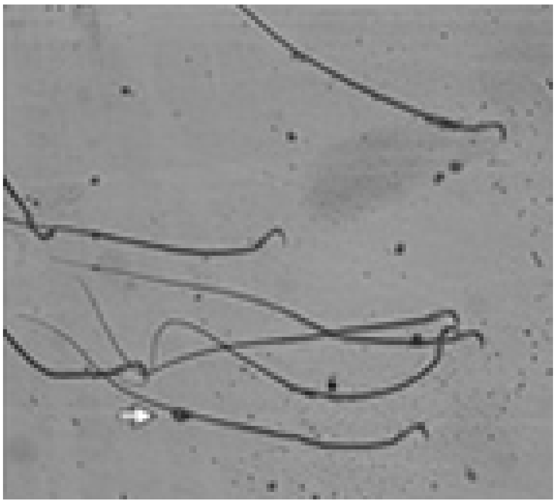

Figure S4. Photographs showing the motility of sperms isolated from rats.
